# Antidepressant-like activity of oroxylin A in mice models of depression: A behavioral and neurobiological characterization

**DOI:** 10.3389/fphar.2022.921553

**Published:** 2022-07-26

**Authors:** Zhong-hua Wu, Hua Fan, Shang-yan Gao, Yan-fei Jin, Cheng chen, Bo Jiang, Jian Shen

**Affiliations:** ^1^ Department of Neurosurgery, The Affiliated Nantong Hospital of Shanghai University (The Sixth People’s Hospital of Nantong), Nantong, China; ^2^ The First Affiliated Hospital, College of Clinical Medicine of Henan University of Science and Technology, Luoyang, China; ^3^ Department of Neurology, The Affiliated Nantong Hospital of Shanghai University (The Sixth People’s Hospital of Nantong), Nantong, China; ^4^ The Affiliated Nantong Hospital of Shanghai University (The Sixth People’s Hospital of Nantong), Nantong, China; ^5^ Department of Pharmacology, School of Pharmacy, Nantong University, Nantong, China; ^6^ Department of Neurosurgery, Rudong People’s Hospital, Nantong, China

**Keywords:** brain-derived neurotrophic factor, chronic restraint stress, chronic unpredictable mild stress, depression, hippocampus, oroxylin A

## Abstract

Depression is a mood disorder which causes a huge economic burden to both families and societies. However, those monoamine-based antidepressants used in clinical practice have been found to have various limitations. Therefore, currently it is very necessary to explore novel antidepressant targets and medications. As a main active component extracted from *Scutellariae radix*, oroxylin A possesses many pharmacological functions such as anti-cancer, anti-inflammation and neuroprotection. Here, the present study aims to investigate whether oroxylin A possess antidepressant-like actions using the chronic unpredictable mild stress (CUMS) and chronic restraint stress (CRS) models of depression, forced swim test, tail suspension test, open field test, sucrose preference test, western blotting, immunofluorescence and viral-mediated gene interference. Our results revealed that treatment of oroxylin A fully prevented both the CUMS-induced and CRS-induced depressive-like behaviors in mice. Moreover, the protecting effects of oroxylin A against CUMS and CRS on mice behaviors were accompanied with a significant enhancement on the levels of brain-derived neurotrophic factor (BDNF), phosphorylated tyrosine kinase B (pTrkB), phosphorylated cAMP-response element binding protein (pCREB) and neurogenesis in the hippocampus. Furthermore, genetic knockdown of BDNF and TrkB in the hippocampus remarkably abolished the antidepressant-like efficacy of oroxylin A in both the CUMS and CRS models of depression, proving that the hippocampal BDNF-TrkB system participates in the antidepressant mechanism of oroxylin A. In summary, our findings are the first evidence showing that oroxylin A possesses potential of being an antidepressant candidate.

## Highlights

1. Treatment of oroxylin A significantly prevented both the CUMS-induced and CRS-induced depressive-like behaviors in mice.
2. Treatment of oroxylin A fully reversed both the CUMS-induced and CRS-induced down-regulation in the BDNF system and neurogenesis in the hippocampus.
3. Genetic knockdown of both BDNF and TrkB in the hippocampus notably abolished the antidepressant-like actions of oroxylin A in mice.

## 1 Introduction

As a mood disorder with high prevalence and recurrence, depression is characterized by a lot of psychiatric and physiological symptoms such as anhedonia, desperate mood, decreased appetite, insomnia and even thoughts of suicide (Nestler et al., 2002; Cui, 2015). It has been reported that by 2030, depression will be the most serious psychiatric disorder in this world (Yang et al., 2015). The pathophysiology of depression is very complex and thought to involve interactions among social, psychological, genetic, and neurobiochemical changes. Antidepressants currently used in clinical practice are thought to produce therapeutic response by enhancing synaptic concentrations of monoamine neurotransmitters such as serotonin (5-HT) and norepinephrine (NA) (López-Muñoz and Alamo, 2009; Pereira and Hiroaki-Sato, 2018). However, these monoamine-based antidepressants have various limitations such as 60% of response rate and 40% of remission rate (Alamo and López-Muñoz, 2009; Blier and El Mansari, 2013; Dale et al., 2015). Therefore, currently it is very necessary to explore novel antidepressant targets and medications.

Neurotrophic factors play a key role in the brain. Brain-derived neurotrophic factor (BDNF) is a widely known neurotrophic factor and critical in antidepressant responses (Neto et al., 2011; Adlam and Zaman, 2013; Castrén and Monteggia, 2021). BDNF has two well-known downstream molecules: tyrosine kinase B (TrkB) and cAMP-response element binding protein (CREB) (Sasi et al., 2017). Similar to BDNF, CREB also play a critical role in the pathophysiology of depression (Blendy, 2006). The neurotrophin factor hypothesis of depression proposes that a decrease of the BDNF-CREB signaling in the hippocampus is the major element triggering depression (Nestler et al., 2002). Moreover, many monoamine-based antidepressants (fluoxetine, paroxetine, venlafaxine, etc.) have also been reported to enhance BDNF biosynthesis after weeks of administration (Yoshimura et al., 2007; Huang et al., 2014; Jin et al., 2017; Li et al., 2017). In addition to depressive-like behaviors and dysfunction in the BDNF system, chronic stress also down-regulates neurogenesis in the dentate gyrus (DG) region of mice (Mahar et al., 2014; Micheli et al., 2018). Hippocampal neurogenesis is modulated by BDNF and required for the therapeutic responses of many antidepressants (Mahar et al., 2014; Micheli et al., 2018). Currently there are no available and acknowledged cellular models of depression, and *in vivo* research is very necessary for exploring novel antidepressants. The chronic unpredictable mild stress (CUMS) model is the most frequently used model of depression in which rodents are intermittently exposed to a series of mild stressors for several weeks, producing a persistent mood state of depression/anxiety in rodents (Qiao et al., 2016; Antoniuk et al., 2019). Similarly, the chronic restraint stress (CRS) model is another well-known and useful model of depression (Qiao et al., 2016; Mao et al., 2022).

Oroxylin A (5′7-dihydroxy-6-methoxy-2phenyl-4H-1-benzopyran-4-one), one of the main active components extracted from *Scutellariae radix*, has already been demonstrated to have a lot of pharmacological functions such as anti-cancer, anti-inflammation, neuroprotection and anti-coagulation (Lu et al., 2016). This substance has now attracted the attention of many researchers. So far several reports have demonstrated a promoting action of oroxylin A on the BDNF-CREB signaling. For example, in 2011, Jeon et al. (2011) reported that oroxylin A treatment increased the production of BDNF in rat primary cortical neuronal culture by activation of the MAPK-CREB pathway. In 2012, Jeon et al. (2012) further revealed that regulation of adenosine A_2_A receptor also underlay the enhancing effects of oroxylin A on the BDNF expression in cortical neurons. Moreover, in 2014, Kim et al. explored the effects of oroxylin A on learning and memory in mice (Kim et al., 2014). Kim et al. found that oroxylin A not only facilitated memory consolidation but also increased the level of hippocampal BDNF in mice (Kim et al., 2014). In addition, many substances which are able to promote BDNF biosynthesis (xanthoceraside, L-701324, 1-methylnicotinamide, etc.) have been reported to protect against depression (Guan et al., 2021; Liu et al., 2021; Zhao et al., 2021). By analyzing these literatures collectively, it is possible that like xanthoceraside, L-701324 and 1-methylnicotinamide, oroxylin A also possesses antidepressant-like efficacy in rodents by enhancing the BDNF system. To confirm this assumption, various methods including the CUMS and CRS models were adopted together to investigate the antidepressant-like actions of oroxylin A in mice comprehensively, with paroxetine used as a positive control. This study would extend the understanding of oroxylin A’s pharmacological activities and may provide a novel antidepressant candidate.

## 2 Materials and methods

### 2.1 Animals

All procedures involving mice were conducted according to the ARRIVE guidelines (Kilkenny et al., 2010; McGrath and Lilley, 2015) and approved by the Animal Welfare Committee of Nantong University. SLAC Laboratory Animal Co., Ltd. (Shanghai, China) provided C57BL/6J mice (male, 8–10 weeks) for this study. Before use, animals were housed (4 per cage, M2 type) under standard conditions (12 h of light/dark cycle, lights on from 07:00 to 19:00 h; (23 ± 1)°C ambient temperature; (55 ± 10)% relative humidity; noise less than 50 dB; ammonia concentration less than 14 mg m^−3^; 24 h of air circulation; and bedding replacement twice a week) for 1 week with ad libitum access to water and rodent chow (Guan et al., 2021; Liu et al., 2021; Zhao et al., 2021). The mice were subjected to stratified randomization according to their body weights. All behavioral experiments were performed in the daytime (between 8:00 am to 5:00 pm). To perform *in vitro* studies, the mice were sacrificed at 9:00 am. For animal sacrifice, the mice were anaesthetized using carbon dioxide and then sacrificed by cervical dislocation. A total of 774 C57BL/6J mice were used for the present study.

### 2.2 Materials

Oroxylin A and paroxetine (the positive control) were purchased from Target Mol (Boston, United States). Adeno-associated virus (AAV)-BDNF-short hairpin RNA (shRNA)-enhanced green fluorescent protein (EGFP), AAV-TrkB-shRNA-EGFP and AAV-Control-shRNA-EGFP were purchased from GeneChem Co., Ltd. (Shanghai, China). The doses of oroxylin A (1, 2, 5 and 10 mg/kg) and paroxetine (20 mg/kg) were determined according to previous studies (Kim et al., 2006; Kim et al., 2007; Kim et al., 2014; Xu et al., 2018). The vehicle for oroxylin A and paroxetine was 0.9% saline containing 10% dimethyl sulfoxide and 20% Cremaphor EL. Oroxylin A and paroxetine were intraperitoneally (i.p., 10 ml/kg) injected. AAV-BDNF-shRNA, AAV-TrkB-shRNA and AAV-Control-shRNA were stereotactically infused into the hippocampus.

### 2.3 Forced swim test

Forced swim test (FST) is a most widely used behavioral assay for detecting potential antidepressants (Petit-Demouliere et al., 2005). As described in several previous reports (Guan et al., 2021; Liu et al., 2021; Zhao et al., 2021), mice were individually placed in a transparent glass tank (45 cm height, 20 cm internal diameter) containing fresh water (15 cm high, 25 ± 1 °C) for a 6-min period. Afterwards, each mouse was dried and returned to its home cage. The immobility duration of each animal in the last 4 min was recorded by an observer unaware of animal grouping. Immobility time was defined as the time spent by the mouse floating in the water without struggling, and making only those movements necessary to keep its head above the water. This test was performed under a light condition.

### 2.4 Tail suspension test

Similar to FST, Tail suspension test (TST) is also widely adopted to screen potential antidepressants (Cryan et al., 2005). As described in several previous reports (Guan et al., 2021; Liu et al., 2021; Zhao et al., 2021), mice were individually glued to a rail 70 cm above the floor by adhesive taps for a 6-min period, and the immobility during the whole period was recorded by an observer unaware of animal grouping. Afterwards, each mouse was returned to its home cage. Mice were considered immobile only when they hung passively and were completely motionless. This test was performed under a light condition.

### 2.5 Open field test

Enhanced lomocotor activity in rodents may contribute to reduction of immobility duration in the FST and TST, leading to false-positive conclusion (Bourin et al., 2001). To exclude this possibility, Open field test (OFT) was used. As described in several previous reports (Guan et al., 2021; Liu et al., 2021; Zhao et al., 2021), mice were individually introduced into an open field apparatus (100 × 100 × 45 cm; 25 squares) for a 5-min period. The apparatus was illuminated with a red bulb (50 W) on the ceiling. The number of peripheral and central squares each animal crossed during the whole period was individually recorded an observer unaware of animal grouping under a dim light environment.

### 2.6 Sucrose preference test

Sucrose preference test (SPT) was done under a light condition, as mentioned in several previous studies (Guan et al., 2021; Liu et al., 2021; Zhao et al., 2021). In brief, the test mice deprived of water for 18 h were individually presented with two bottles: one filled with 1% sucrose solution and the other with tap water. The testing period lasted for 6 h and the consumption of liquids were determined by subtracting the bottle weights. Before the test, a 48-h sucrose preference training procedure has been performed for each animal. Sucrose preference (%) was calculated as sucrose solution consumption divided by total fluid consumption, multiplied by 100.

### 2.7 Chronic unpredictable mild stress

Chronic unpredictable mild stress (CUMS) was done as mentioned in several previous studies (Gao et al., 2020; Wang et al., 2020; Tang et al., 2022). Briefly, eight stressors were adopted in the present study: damp bedding (24 h), cage tilting (12 h), restraint (1 h), shaking (30 min), 4°C exposure (1 h), day/night inversion, food deprivation (23 h) or water deprivation (23 h). The whole CUMS period lasted for 8 weeks, and administration of oroxylinA/paroxetine/vehicle was performed daily in the last 2 weeks. Mice in the control group were handled daily. After CUMS, the FST, TST and sucrose preference test (SPT) were adopted together to assay the depressive-like behaviors of mice.

### 2.8 Chronic restraint stress

Chronic restraint stress (CRS) was done as mentioned in several previous studies (Gao et al., 2020; Wang et al., 2020; Tang et al., 2022). Briefly, mice in the stressed groups were individually subjected to 8 weeks of CRS (3 h/d, 9:00 a.m. to 12:00 a.m.), and administration of oroxylinA/paroxetine/vehicle was performed daily in the last 2 weeks. Conical plastic tubes (containing vent holes) of 50 ml were used for restraint stress. Mice in the control group were handled daily. The depressive-like behaviors of mice were assayed using the FST, TST and SPT.

### 2.9 Western blotting

This method was done as we described before (Wu et al., 2018; Chen et al., 2019; Gao et al., 2022). Protein samples of 30 μg were loaded on 10/12% SDS-PAGE gels (Beyotime, Shanghai, China). The western blotting procedures were performed in a commonly adopted manner: 1, SDS-PAGE separation; 2, proteins transfer; 3, proteins blocking; 4, TBST washing; 5, primary antibodies incubation; 6, TBST washing; 7, secondary antibodies incubation; 8, TBST washing; 9, membranes scanning. Primary antibodies against BDNF (1:500; Abcam, Bristol, United Kingdom), TrkB (1:1000; Abcam), phospho-TrkB-Tyr515 (pTrkB; 1:500; Abcam), CREB (1:1000; Cell signaling, Danvers, United States), phospho-CREB-Ser133 (pCREB; 1:500; Cell signaling) and β-actin (1:2000; Cell signaling) were used. IR-Dye 680-labeled secondary antibodies (1:5000; Licor, Lincoln, United States) were also used. An Odyssey CLx detection system was adopted for membranes scanning.

### 2.10 Immunofluorescence

In the present study, the level of hippocampal neurogenesis in mice was determined by doublecortin (DCX) immunofluorescence, as mentioned in two previous studies (Jiang et al., 2019; Guan et al., 2021). In brief, mice anesthetized with 0.5% sodium pentobarbital were subjected to transcardial perfusion of 4%paraformaldehyde. After post-fixation and dehydration, 25 μm of hippocampal slices were collected. For DCX staining, the slices were dealt in a commonly adopted manner: 1, 0.3% Triton X-100 incubation; 2, 3% BSA incubation; 3, primary antibody incubation; 4, washed in PBS; 5, secondary antibody incubation; 6, washed in PBS; 7, 4’,6-diamidino-2-phenylindole (DAPI) incubation; 8, washed in PBS; 9, coverslipped and observed. Primary antibody against DCX (1:100; Cell signaling) and fluorescein isothiocyanate (FITC)-labeled secondary antibody (1:50; Thermo Fisher, Waltham, United States) were used. The method of DCX examination has been described as described before (Jiang et al., 2019).

### 2.11 AAV-mediated genetic knockdown of BDNF and TrkB in the hippocampus

The production of AAV-BDNF-shRNA-EGFP, AAV-TrkB-shRNA-EGFP and AAV-Control-shRNA-EGFP has already been described in a previous report (Jiang et al., 2019). In this study, each mouse anesthetized with 0.5% pentobarbital sodium was fixed in a stereotactic frame (Stoelting, Wood Dale, United States). After cutting the scalp, the skull of each mouse was exposed using 75% ethanol and 1% H_2_O_2_. Two small drill holes were bilaterally made on the skull, and then, a 10 μl Hamilton syringe was placed at the hippocampus coordinates: AP =−2.3 mm, ML = ± 1.6 mm, DV = + 1.8 mm. AAV-BDNF-shRNA, AAV-TrkB-shRNA or AAV-Control-shRNA was bilaterally infused into the hippocampus region of each mouse using the syringe at a rate of 0.5 μl/min (1.5 μl/each side). Afterwards, 5 min of waiting was needed to prevent AAV reflux. The wound of each mouse was cleaned and sutured. A period of 2 weeks was required for the expression of AAV to be stable in the hippocampus. Furthermore, these animals were subjected to 8 weeks of chronic stress (CUMS or CRS) and another 2 weeks of oroxylin A/vehicle administration, followed by the FST, TST and SPT.

All AAVs were adjusted to 5 × 10^12^ TU/ml before use. The nucleotide sequences for BDNF-shRNA, TrkB-shRNA and Control-shRNA were 5′-TGAGCGTGTGTGAC AGTATTA-3′, 5′-GCA​ACC​TGC​GGC​ACA​TAA​A-3′ and 5′-TTCTCCGAACGTGTC ACGT-3′, respectively (Jiang et al., 2019).

### 2.12 Experimental protocols

#### 2.12.1 Experiment 1

This experiment aimed to preliminarily assay the antidepressant-like potential of oroxylin A in mice (216 mice were used). Briefly, naive mice received a single injection of vehicle/paroxetine (20 mg/kg)/Oroxylin A (1, 2, 5 or 10 mg/kg). After 30 min, the FST, TST or OFT was performed. Separate groups of mice were used for the three tests (*n* = 12).

#### 2.12.2 Experiment 2

This experiment was performed to determine the antidepressant-like actions of oroxylin A in mice using CUMS (96 mice were used). Briefly, naive mice were subjected to 8 weeks of CUMS and received a daily injection of vehicle/paroxetine (20 mg/kg)/oroxylin A (2 or 5 mg/kg) during the final 2 weeks (*n* = 12). Then, the FST, TST, and SPT were performed successively. Furthermore, mice from each group were randomly selected for western blot (*n* = 6) and immunofluorescence (*n* = 6) assays.

#### 2.12.3 Experiments 3

This experiment was performed to determine the antidepressant-like actions of oroxylin A in mice using CRS (96 mice were used). Briefly, naive mice were subjected to 8 weeks of CRS and received a daily injection of vehicle/paroxetine (20 mg/kg)/oroxylin A (2 or 5 mg/kg) during the final 2 weeks (*n* = 12). Then, the FST, TST, and SPT were performed successively. Furthermore, mice from each group were randomly selected for western blot (*n* = 6) and immunofluorescence (*n* = 6) assays.

#### 2.12.4 Experiments 4

This experiment was performed to explore the antidepressant-like mechanism of oroxylin A in mice using CUMS and BDNF-shRNA (99 mice were used). Naive mice infused with Control-shRNA/BDNF-shRNA were housed for 14 days and then subjected to 8 weeks of CUMS and 2 weeks of vehicle/oroxylin A (5 mg/kg) administration (*n* = 12). Afterwards, the FST, TST and SPT were performed successively.

#### 2.12.5 Experiments 5

This experiment was performed to explore the antidepressant-like mechanism of oroxylin A in mice using CRS and BDNF-shRNA (84 mice were used). Naive mice infused with Control-shRNA/BDNF-shRNA were housed for 14 days and then subjected to 8 weeks of CRS and 2 weeks of vehicle/oroxylin A (5 mg/kg) administration (*n* = 12). Afterwards, the FST, TST and SPT were performed successively.

#### 2.12.6 Experiments 6

This experiment was performed to explore the antidepressant-like mechanism of oroxylin A in mice using CUMS and TrkB-shRNA (99 mice were used). Naive mice infused with Control-shRNA/TrkB-shRNA were housed for 14 days and then subjected to 8 weeks of CUMS and 2 weeks of vehicle/oroxylin A (5 mg/kg) administration (*n* = 12). Afterwards, the FST, TST, and SPT were performed successively.

#### 2.12.7 Experiments 7

This experiment was performed to explore the antidepressant-like mechanism of oroxylin A in mice using CRS and TrkB-shRNA (84 mice were used). Naive mice infused with Control-shRNA/TrkB-shRNA were housed for 14 days and then subjected to 8 weeks of CRS and 2 weeks of vehicle/oroxylin A (5 mg/kg) administration (*n* = 12). Afterwards, the FST, TST, and SPT were performed successively.

### 2.13 Statistical analysis

Statistical analyses in the present study were done using the SPSS 26.0 software (SPSS Inc., Chicago, United States). Multiple group comparisons were achieved by (one-way analysis of variance (ANOVA) + Tukey’s test) or (two-way ANONA + Bonferroni’s test). All data are displayed as means ± standard error of the mean (S.E.M.). *p* < 0.05 was regarded as statistically significant.

## 3 Results

### 3.1 FST, TST and OFT were used together to preliminarily detect the antidepressant potential of oroxylin A in naive mice

**FIGURE 1:**
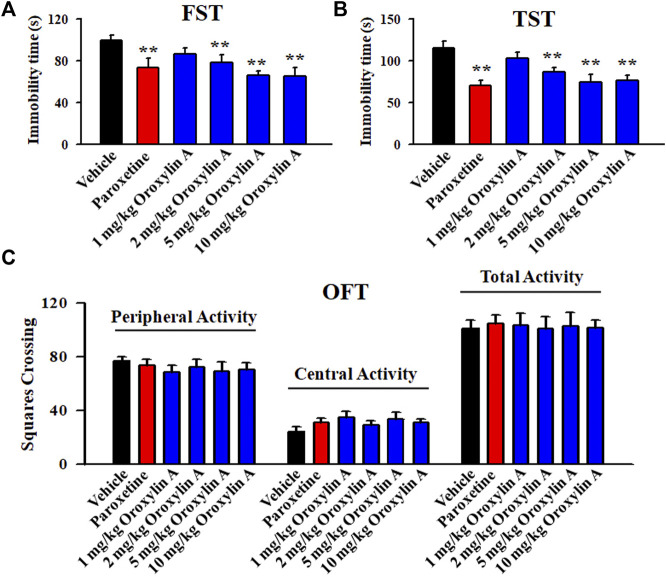
Oroxylin A exhibited potential of being an antidepressant candidate in the FST and TST. A single treatment of vehicle, paroxetine (20 mg/kg) or oroxylin A (1, 2, 5 and 10 mg/kg) was given to naïve C57BL/6J mice, and after 30 min, the FST, TST or OFT was conducted. **(A)** Oroxylin A and paroxetine significantly reduced the immobility duration of naive mice in the FST. **(B)** Oroxylin A and paroxetine notably decreased the immobility duration of naive mice in the TST. **(C)** Neither oroxylin A nor paroxetine induced significant effects on the locomotor activity of naive mice in the OFT. All data are displayed as means ± S.E.M. (*n* = 12); ^**^ *p <* 0.01 vs. Vehicle. The statistical comparisons were achieved by (One-way ANOVA + Tukey’s test).

As to whether oroxylin A has antidepressant potential, the FST and TST were firstly adopted. Figure 1A illustrates the FST data and shows that the immobility duration of mice in the vehicle-treated group was significantly longer than that of mice in the paroxetine-treated and oroxylin A-treated groups (*n* = 12, *p* < 0.01). Detailed data analysis reveals that compared with the vehicle, 2 and 5 mg/kg oroxylin A treatment induced a 21.6 ± 3.15% and 33.9 ± 6.22% decrease in the FST immobility, respectively. The effects of 10 mg/kg oroxylin A were comparable to those of 5 mg/kg oroxylin A and 20 mg/kg paroxetine. ANOVA analysis indicates a notable effect of drug treatment [F (5, 66) = 26.345, *p* < 0.01]. Figure 1B illustrates the TST data and shows that administration of 2 and 5 mg/kg oroxylin A induced a 24.4 ± 5.36% and 35.1 ± 4.88% reduction in the TST immobility, respectively (*n* = 12, *p* < 0.01). The effects of 10 mg/kg oroxylin A were nearly the same with those of 5 mg/kg oroxylin A and 20 mg/kg paroxetine. ANOVA analysis also reveals a notable effect of drug treatment [F (5, 66) = 32.427, *p* < 0.01]. Therefore, 2 and 5 mg/kg were chosen as the doses of oroxylin A in the following studies.

Moreover, the OFT results reveal no significant effects of oroxylin A on mice locomotor activity. No significant differences among the groups in the number of squares that a mouse crossed in the central or peripheral area were found (*n* = 12; Figure 1C). Correspondingly, ANOVA analysis shows no effects of drug treatments [F (5, 66) = 1.859, *p* = 0.236]. Collectively, oroxylin A possesses antidepressant potential in mice.

### 3.2 Repeated oroxylin A treatment reversed both the CUMS-induced and CRS-induced depressive-like behaviors in mice

**FIGURE 2:**
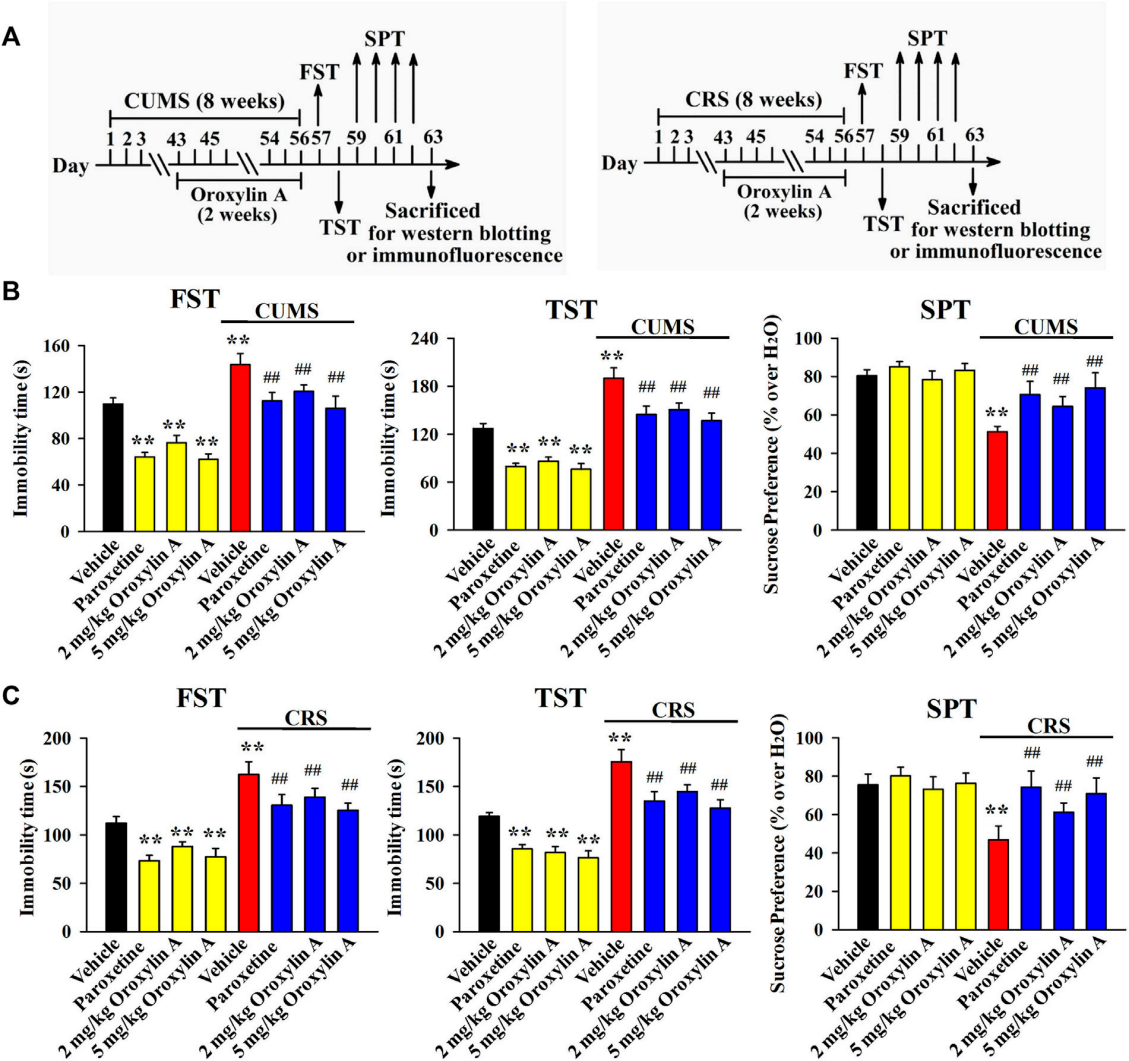
Administration of oroxylin A notably ameliorated both the CUMS-induced and CRS-induced behavioral symptoms in mice. **(A)** Two schematics respectively showing the timeline of the experimental procedures involving CUMS and CRS. **(B)** Mice subjected to CUMS were given daily administration of vehicle, paroxetine (20 mg/kg) or oroxylin A (2 and 5 mg/kg) during the last 2 weeks, and afterwards, behavioral tests were performed. Mice in the (CUMS + oroxylin A)-treated and (CUMS + paroxetine)-treated groups spent significantly less time being immobile than mice in the (CUMS + vehicle)-treated group in the FST and TST. Also, mice in the (CUMS + oroxylin A)-treated and (CUMS + paroxetine)-treated groups displayed notably higher sucrose preference than mice in the (CUMS + vehicle)-treated group. **(C)** Mice subjected to CRS were given daily administration of vehicle, paroxetine (20 mg/kg) or oroxylin A (2 and 5 mg/kg) during the last 2 weeks, and afterwards, behavioral tests were performed. Mice in the (CRS + oroxylin A)-treated and (CRS + paroxetine)-treated groups had significantly less immobility than mice in the (CRS + vehicle)-treated group in the FST and TST. Also, mice in the (CRS + oroxylin A)-treated and (CRS + paroxetine)-treated groups exhibited evidently higher sucrose preference than mice in the (CRS + vehicle)-treated group. All data are displayed as means ± S.E.M. (*n* = 12); ^**^ *p* < 0.01 vs. Vehicle; ^##^ *p <* 0.01 vs. (CUMS + Vehicle) or (CRS + Vehicle). The statistical comparisons were achieved by (Two-way ANOVA + Bonferroni’s test).

Then, the CUMS model of depression was established. As shown in Figure 2B, mice in the CUMS group had remarkably more immobility in the FST and TST than mice in the vehicle-treated control group (*n* = 12, *p* < 0.01), while repeated treatment of both paroxetine and oroxylin A evidently prevented the promoting effects of CUMS on mice immobility in the FST [ANOVA: CUMS, F (1, 88) = 28.647, *p* < 0.01; Drug, F (3, 88) = 23.119, *p* < 0.01; Interaction, F (3, 88) = 16.239, *p* < 0.01] and TST [ANOVA: CUMS, F (1, 88) = 29.835, *p* < 0.01; Drug, F (3, 88) = 20.942, *p* < 0.01; Interaction, F (3, 88) = 14.663, *p* < 0.01] (*n* = 12, *p* < 0.01). Detailed data analysis shows that CUMS exposure enhanced the immobility of mice in the FST and TST by 30.9 ± 4.15% and 49.3 ± 7.46%, respectively. In contrast, the FST immobility of the CUMS-exposed mice was decreased by 16 ± 3.31% and 26.2 ± 5.14% under treatment of 2 and 5 mg/kg oroxylin A, respectively. The TST immobility of the CUMS-exposed mice was decreased by 20.7 ± 4.65% and 28 ± 6.04% under treatment of 2 and 5 mg/kg oroxylin A, respectively. The SPT results are illustrated in Figure 2B [ANOVA: CUMS, F (1, 88) = 21.667, *p* < 0.01; Drug, F (3, 88) = 17.607, *p* < 0.01; Interaction, F (3, 88) = 11.223, *p* < 0.01]. Exposure to CUMS led to a 36.3 ± 4.72% decrease in the sucrose preference of mice compared with that of the vehicle-treated control mice (*n* = 12, *p* < 0.01), and this behavioral change was notably reversed by administration of paroxetine and oroxylin A (*n* = 12, *p* < 0.01). Detailed data analysis shows that the sucrose preference of mice in the CUMS group was enhanced by 25.8 ± 5.05% and 44.7 ± 7.38% under treatment of 2 and 5 mg/kg oroxylin A, respectively.

\Moreover, the CRS model of depression was also employed, and the behavioral results were similar to CUMS. It was found that administration of paroxetine and oroxylin A for 2 weeks notably ameliorated the promoting effects of CRS on the immobility of mice in the FST [ANOVA: CRS, F (1, 88) = 33.408, *p* < 0.01; Drug, F (3, 88) = 25.154, *p* < 0.01; Interaction, F (3, 88) = 19.758, *p* < 0.01; Figure 2C] and TST [ANOVA: CRS, F (1, 88) = 27.825, *p* < 0.01; Drug, F (3, 88) = 23.291, *p* < 0.01; Interaction, F (3, 88) = 16.007, *p* < 0.01; Figure 2C] as well as the decreasing effects of CRS on the sucrose preference of mice [ANOVA: CRS, F (1, 88) = 22.404, *p* < 0.01; Drug, F (3, 88) = 13.779, *p* < 0.01; Interaction, F (3, 88) = 10.709, *p* < 0.01; Figure 2C] (*n* = 12, *p* < 0.01). Detailed data analysis reveals that CRS exposure enhanced the immobility of mice in the FST and TST by 44.8 ± 8.37% and 47.2 ± 6.89%, respectively. CRS exposure also induced a 38 ± 5.52% decrease in the sucrose preference of mice. In contrast, the FST immobility of the CRS-exposed mice was decreased by 14.4 ± 4.03% and 22.7 ± 6.24% under treatment of 2 and 5 mg/kg oroxylin A, respectively. The TST immobility of the CRS-exposed mice was decreased by 17.5 ± 3.72% and 27.3 ± 5.19% under treatment of 2 and 5 mg/kg oroxylin A, respectively. The sucrose preference of the CRS-exposed mice was increased by 31 ± 6.34% and 51.5 ± 8.91% under treatment of 2 and 5 mg/kg oroxylin A, respectively. Taken together, oroxylin A indeed has antidepressant-like efficacy in mice.

### 3.3 Repeated oroxylin A treatment prevented both the CUMS-induced and CRS-induced dysfunction in the hippocampal BDNF signaling cascade and neurogenesis

**FIGURE 3:**
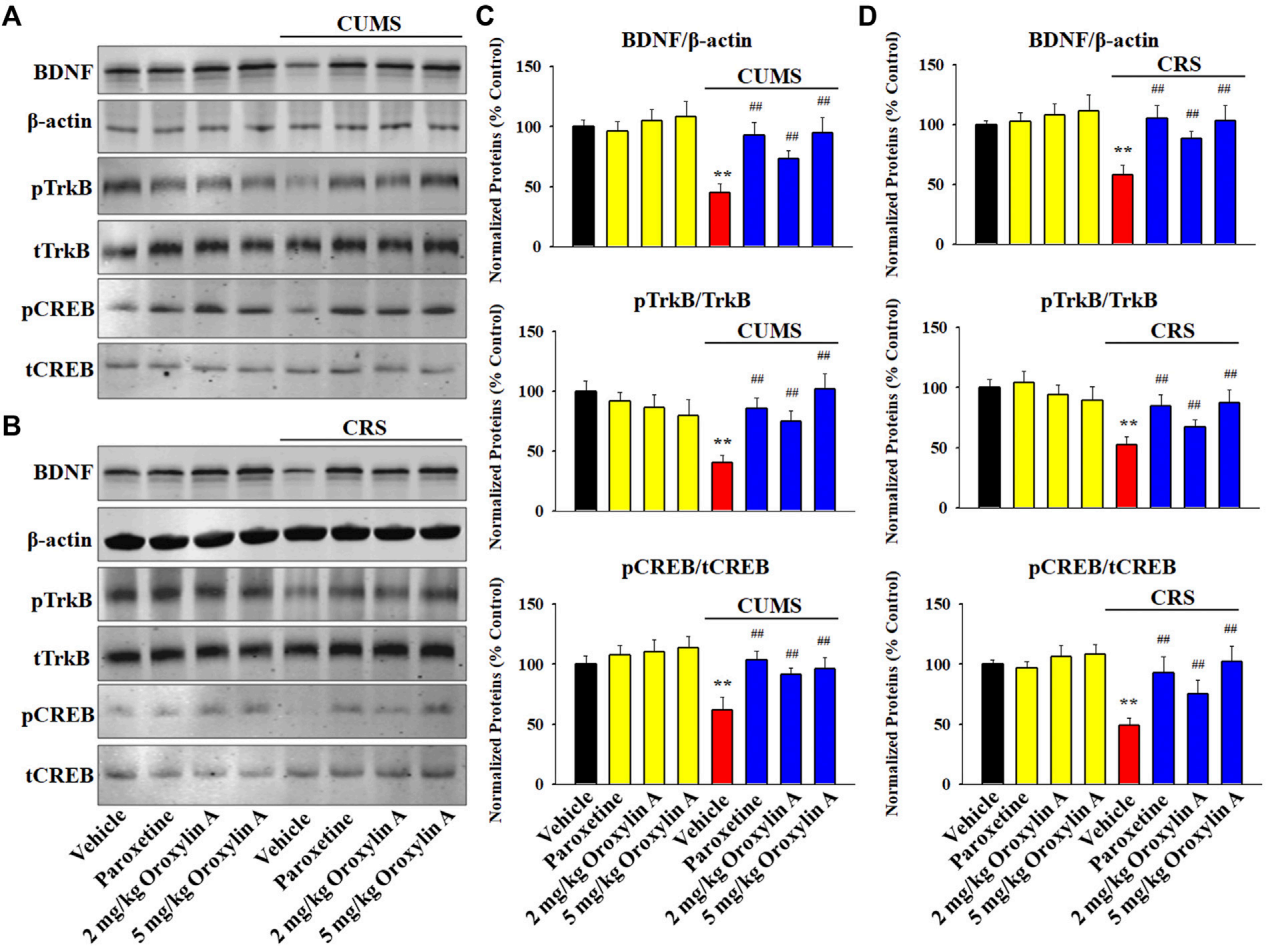
Administration of oroxylin A enhanced the hippocampal BDNF signaling cascade in both the CUMS-treated and CRS-treated mice. **(A** and **C)** Representative western blotting images and corresponding analyses show that mice in the (CUMS + oroxylin A)-treated and (CUMS + paroxetine)-treated groups had evidently higher protein levels of BDNF, pTrkB and pCREB in the hippocampus than mice in the (CUMS + vehicle)-treated group. **(B** and **D)** Representative images of western blotting and corresponding analyses indicated that mice in the (CRS + oroxylin A)-treated and (CRS + paroxetine)-treated groups also had remarkably higher protein levels of BDNF, pTrkB and pCREB in the hippocampus than mice in the (CRS + vehicle)-treated group. All data are displayed as means ± S.E.M. (*n* = 6); ^**^ *p* < 0.01 vs. Vehicle; ^##^ *p <* 0.01 vs. (CUMS + Vehicle) or (CRS + Vehicle). The statistical comparisons were achieved by (Two-way ANOVA + Bonferroni’s test).

Western blotting was adopted to examine the expression of the hippocampal BDNF signaling cascade following chronic stress and drug treatments. The results involving CUMS are displayed in Figures 3A,C. It was found that repeated treatment of both paroxetine and oroxylin A fully reversed the down-regulating effects of CUMS on the protein levels of BDNF [ANOVA: CUMS, F (1, 40) = 28.114, *p* < 0.01; Drug, F (3, 40) = 22.379, *p* < 0.01; Interaction, F (3, 40) = 15.406, *p* < 0.01], pTrkB [ANOVA: CUMS, F (1, 40) = 34.557, *p* < 0.01; Drug, F (3, 40) = 28.413, *p* < 0.01; Interaction, F (3, 40) = 21.264, *p* < 0.01] and pCREB [ANOVA: CUMS, F (1, 40) = 25.901, *p* < 0.01; Drug, F (3, 40) = 19.718, *p* < 0.01; Interaction, F (3, 40) = 14.336, *p* < 0.01] in the hippocampus of mice (n = 6, *p* < 0.01). The results involving CRS are displayed in Figures 3B,D. In parallel with the CUMS data, administration of paroxetine and oroxylin A notably ameliorated the CRS-induced decreasing effects on the protein levels of BDNF [ANOVA: CRS, F (1, 40) = 26.719, *p* < 0.01; Drug, F (3, 40) = 22.773, *p* < 0.01; Interaction, F (3, 40) = 18.675, *p* < 0.01], pTrkB [ANOVA: CRS, F (1, 40) = 30.149, *p* < 0.01; Drug, F (3, 40) = 21.665, *p* < 0.01; Interaction, F (3, 40) = 13.887, *p* < 0.01] and pCREB [ANOVA: CRS, F (1, 40) = 24.205, *p* < 0.01; Drug, F (3, 40) = 18.992, *p* < 0.01; Interaction, F (3, 40) = 12.639, *p* < 0.01] in the hippocampus (*n* = 6, *p* < 0.01). In addition, administration of oroxylin A did not influence the protein levels of hippocampal BDNF, pTrkB and pCREB in naïve control mice (*n* = 6). The protein expression of total TrkB, CREB and β-actin were unchanged among all groups.

**FIGURE 4:**
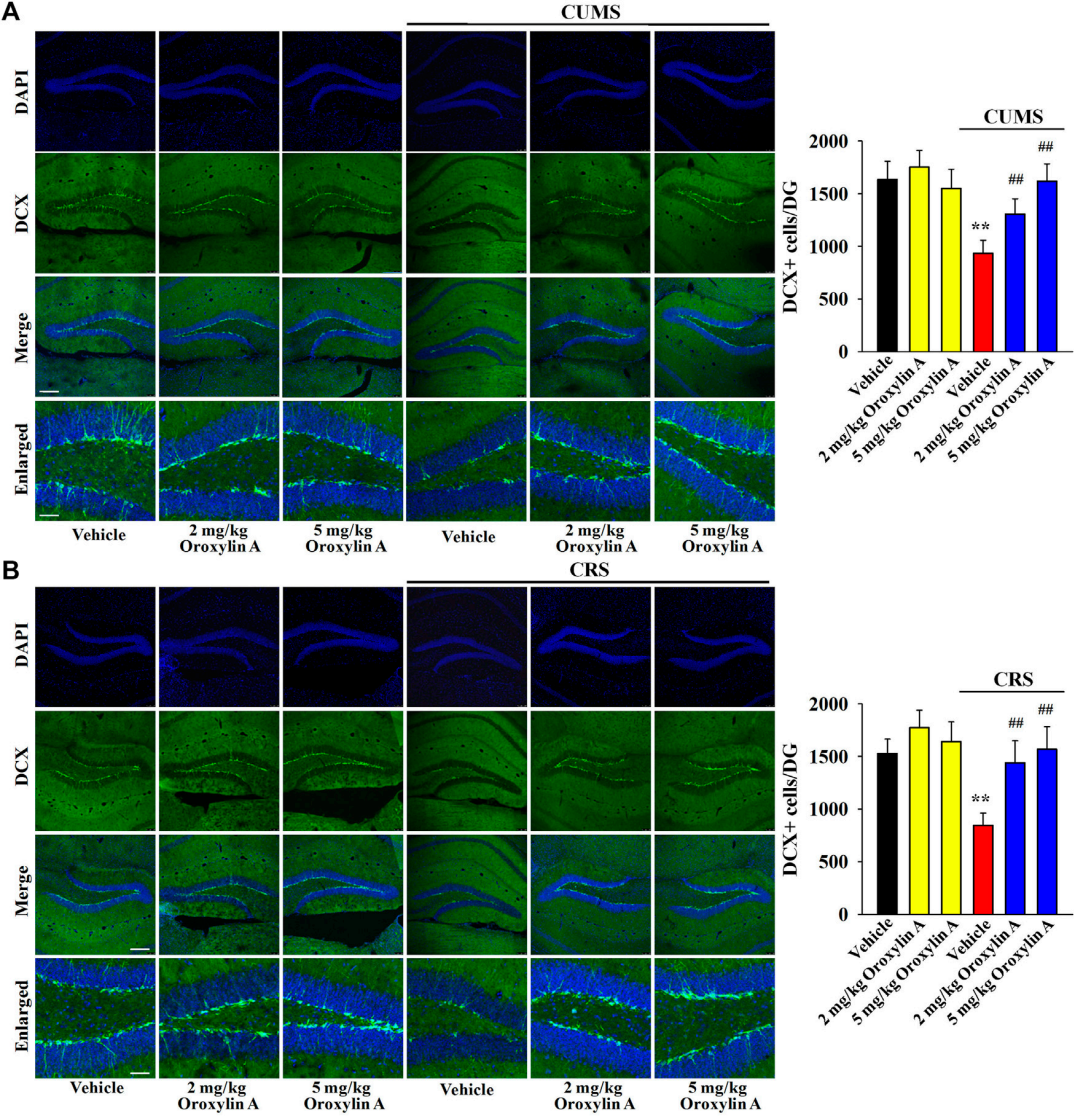
The levels of hippocampal neurogenesis in both the CUMS-treated and CRS-treated mice were promoted by administration of oroxylin A. **(A)** Representative images of confocal microscopy and corresponding analyses revealed that mice in the (CUMS + oroxylin A)-treated and (CUMS + paroxetine)-treated groups exhibited evidently more DCX^+^ cells than mice in the (CUMS + vehicle)-treated group. The scale bars of representative and enlarged images are 150 and 37.5 μm, respectively. **(B)** Representative images of confocal microscopy and corresponding analyses indicated that mice in the (CRS + oroxylin A)-treated and (CRS + paroxetine)-treated groups had notably more DCX^+^ cells than mice in the (CRS + vehicle)-treated group. The scale bars of representative and enlarged images are 150 and 37.5 μm, respectively. All data are displayed as means ± S.E.M. (*n* = 6); ^**^ *p* < 0.01 vs. Vehicle; ^##^ *p <* 0.01 vs. (CUMS + Vehicle) or (CRS + Vehicle). The statistical comparisons were achieved by (Two-way ANOVA + Bonferroni’s test).

DCX immunofluorescence in the DG region was performed to examine whether oroxylin A administration can prevent the down-regulating effects of chronic stress on hippocampal neurogenesis. Figure 4A shows the immunofluorescence data involving CUMS. Exposure to CUMS led to a 42.9 ± 8.28% reduction in the DCX^+^ cells amount in the DG, while oroxylin A administration completely reversed this change [ANOVA: CUMS, F (1, 40) = 42.815, *p* < 0.01; Drug, F (3, 40) = 36.558, *p* < 0.01; Interaction, F (3, 40) = 27.206, *p* < 0.01] (*n* = 6, *p* < 0.01). Figure 4B reveals the immunofluorescence data involving CRS. Similarly, exposure to CRS led to a 44.7 ± 6.59% reduction in the DCX^+^ cells amount in the DG, while oroxylin A administration evidently prevented this effect [ANOVA: CRS, F (1, 40) = 45.611, *p* < 0.01; Drug, F (3, 40) = 35.105, *p* < 0.01; Interaction, F (3, 40) = 23.489, *p* < 0.01] (*n* = 6, *p* < 0.01). Moreover, oroxylin A treatment did not affect the DCX^+^ cells amount in the DG of naïve control mice (*n* = 6).

In summary, oroxylin A has beneficial effects against the chronic stress-induced dysfunction in the hippocampal BDNF system and neurogenesis.

### 3.4 Hippocampal BDNF-TrkB system is necessary for the antidepressant-like actions of oroxylin A in the CUMS and CRS models of depression

**FIGURE 5:**
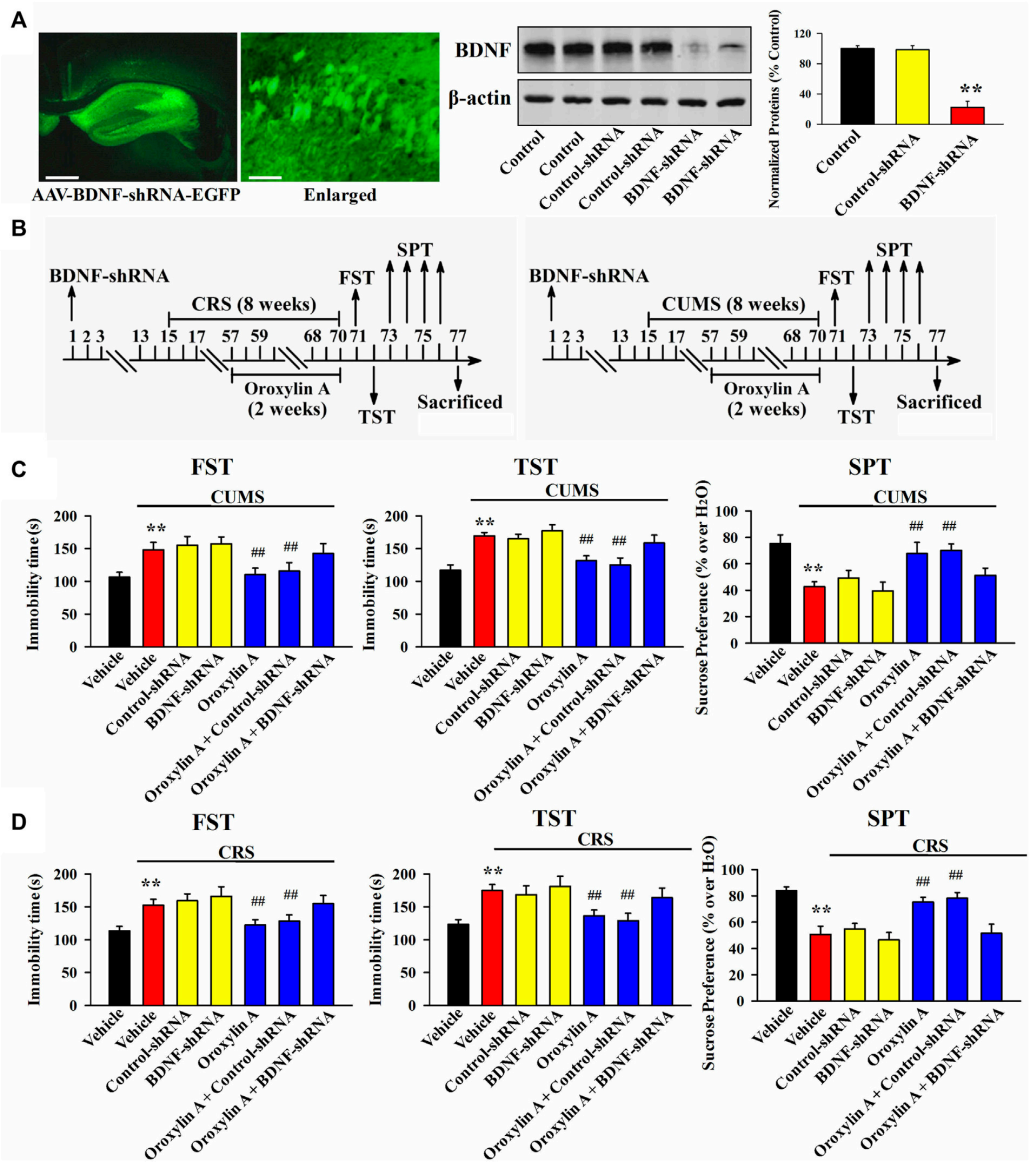
Hippocampal BDNF-knockdown by BDNF-shRNA abrogated the antidepressant activity of oroxylin A in mice. **(A)** Fluorescence images of a fixed hippocampal slice which expressed AAV-BDNF-shRNA-EGFP 2 weeks after its stereotactic infusion. The scale bars of representative and enlarged images are 400 and 50 μm, respectively. The following western blotting results confirmed the silencing effects of BDNF-shRNA on the protein expression of hippocampal BDNF (*n* = 5). **(B)** Two schematics respectively showing the timeline of the experimental procedures involving CUMS and CRS. **(C)** Mice in the (CUMS + oroxylin A + BDNF-shRNA)-treated group spent significantly more time being immobile than mice in the (CUMS + oroxylin A)-treated and (CUMS + oroxylin A + Control-shRNA)-treated groups in the FST and TST (*n* = 12). Also, mice in the (CUMS + oroxylin A + BDNF-shRNA)-treated group displayed notably lower sucrose preference than mice in the (CUMS + oroxylin A)-treated and (CUMS + oroxylin A + Control-shRNA)-treated groups (n = 12). **(D)** Mice in the (CRS + oroxylin A + BDNF-shRNA)-treated group had significantly more immobility than mice in the (CRS + oroxylin A)-treated and (CRS + oroxylin A + Control-shRNA)-treated groups in the FST and TST (*n* = 12). Also, mice in the (CRS + oroxylin A + BDNF-shRNA)-treated group exhibited evidently lower sucrose preference than mice in the (CRS + oroxylin A)-treated and (CRS + oroxylin A + Control-shRNA)-treated groups (*n* = 12). All data were displayed as means ± S.E.M; ^**^ *p* < 0.01 vs. Vehicle; ^##^ *p <* 0.01 vs. (CUMS + Vehicle) or (CRS + Vehicle). The statistical comparisons were achieved by (One-way ANOVA + Tukey’s test).

**FIGURE 6:**
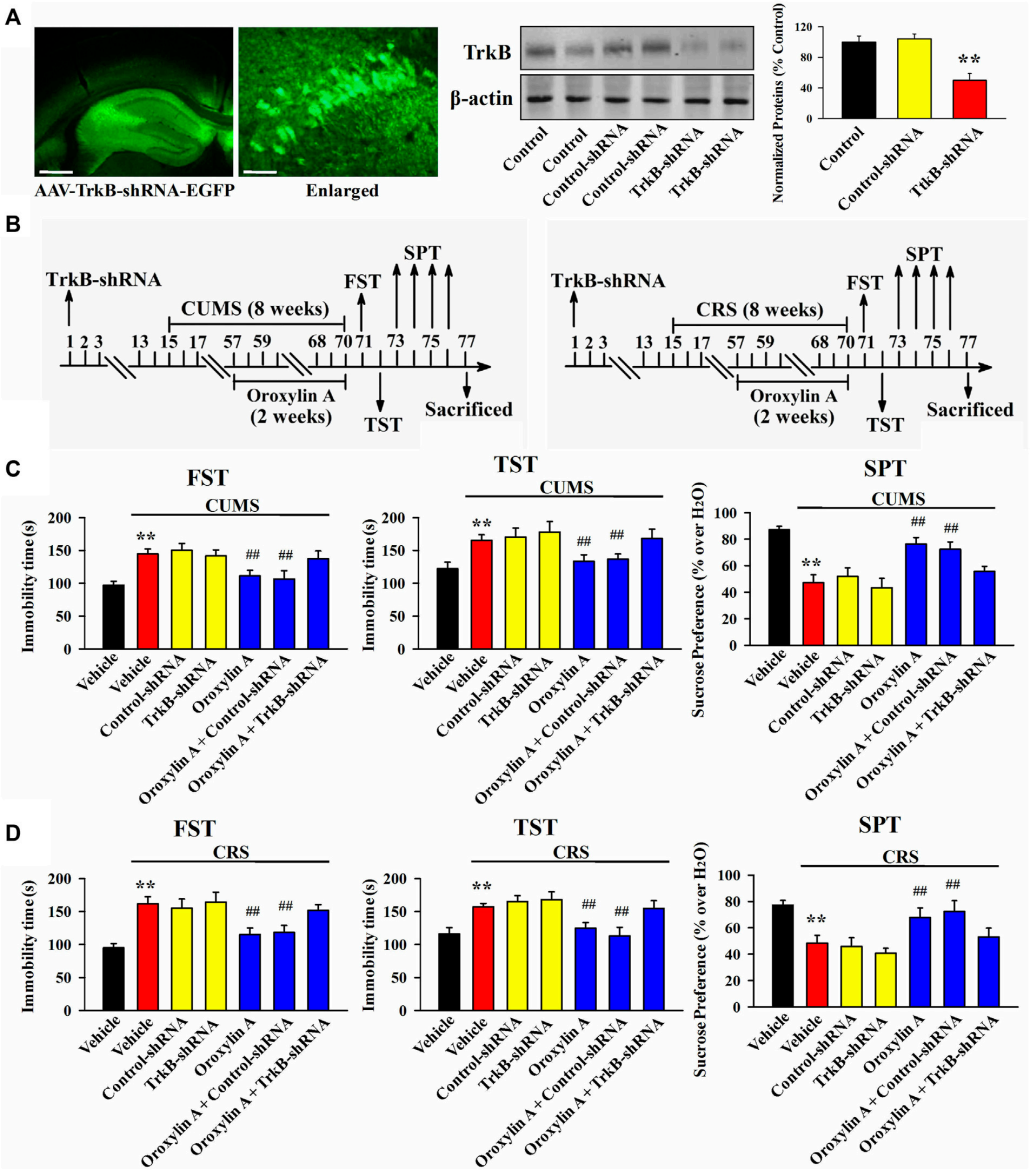
Hippocampal TrkB-knockdown by TrkB-shRNA abolished the antidepressant activity of oroxylin A in mice. **(A)** Fluorescence images of a fixed hippocampus slice which expressed AAV-TrkB-shRNA-EGFP 2 weeks after its stereotactic infusion. The scale bars of representative and enlarged images are 400 and 50 μm, respectively. The following western blotting results confirmed the silencing effects of TrkB-shRNA on the protein expression of hippocampal TrkB (*n* = 5). **(B)** Two schematics respectively showing the timeline of the experimental procedures involving CUMS and CRS. **(C)** Mice in the (CUMS + oroxylin A + TrkB-shRNA)-treated group spent significantly more time being immobile than mice in the (CUMS + oroxylin A)-treated and (CUMS + oroxylin A + Control-shRNA)-treated groups in the FST and TST (*n* = 12). Also, mice in the (CUMS + oroxylin A + TrkB-shRNA)-treated group displayed notably lower sucrose preference than mice in the (CUMS + oroxylin A)-treated and (CUMS + oroxylin A + Control-shRNA)-treated groups (*n* = 12). **(D)** Mice in the (CRS + oroxylin A + TrkB-shRNA)-treated group had significantly more immobility than mice in the (CRS + oroxylin A)-treated and (CRS + oroxylin A + Control-shRNA)-treated groups in the FST and TST (*n* = 12). Also, mice in the (CRS + oroxylin A + TrkB-shRNA)-treated group exhibited evidently lower sucrose preference than mice in the (CRS + oroxylin A)-treated and (CRS + oroxylin A + Control-shRNA)-treated groups (*n* = 12). All data were displayed as means ± S.E.M; ^**^ *p* < 0.01 vs. Vehicle; ^##^ *p <* 0.01 vs. (CUMS + Vehicle) or (CRS + Vehicle). The statistical comparisons were achieved by (One-way ANOVA + Tukey’s test).

From the above results it can be speculated that oroxylin A may produce antidepressant-like efficacy by promoting the hippocampal BDNF-TrkB system. To validate this assumption, AAV-BDNF-shRNA-EGFP and AAV-TrkB-shRNA-EGFP were respectively used to selectively knockdown the hippocampal expression of BDNF and TrkB. As shown in Figure 5A, Figure6A, AAV was stably expressed in the hippocampal neurons 2 weeks after stereotactic infusion, and the silencing efficacy of BDNF-shRNA and TrkB-shRNA were confirmed (*n* = 5, *p* < 0.01). In brief, mice infused with BDNF-shRNA or TrkB-shRNA were maintained for 2 weeks and then subjected to chronic stress (CUMS or CRS) and 5 mg/kg oroxylin A treatment. Afterwards, the behavioral tests were performed (Figure 5B, Figure 6B).

Figure 5C shows the results involving CUMS and BDNF-shRNA. It can be found that BDNF-shRNA infusion remarkably blocked the decreasing effects of 5 mg/kg oroxylin A administration on the FST immobility [ANOVA: F (6, 77) = 37.645, *p* < 0.01] and TST immobility [ANOVA: F (6, 77) = 32.883, *p* < 0.01] of mice subjected to CUMS (*n* = 12, *p* < 0.01). Additionally, BDNF-shRNA infusion remarkably blocked the increasing effects of 5 mg/kg oroxylin A administration on the sucrose preference of mice subjected to CUMS [ANOVA: F (6, 77) = 24.227, *p* < 0.01] (n = 12, *p* < 0.01). Figure 5D shows the results involving CRS and BDNF-shRNA. The usage of BDNF-shRNA evidently prevented the decreasing effects of oroxylin A on the FST immobility [ANOVA: F (6, 77) = 29.071, *p* < 0.01] and TST immobility [ANOVA: F (6, 77) = 26.547, *p* < 0.01] of mice subjected to CRS (*n* = 12, *p* < 0.01). Moreover, the usage of BDNF-shRNA evidently prevented the enhancing effects of oroxylin A on the sucrose preference of mice subjected to CRS [ANOVA: F (6, 77) = 17.662, *p* < 0.01] (*n* = 12, *p* < 0.01).

Figure 6C illustrates the data involving CUMS and TrkB-shRNA. It can be found that TrkB-shRNA infusion remarkably blocked the decreasing effects of 5 mg/kg oroxylin A administration on the FST immobility [ANOVA: F (6, 77) = 28.125, *p* < 0.01] and TST immobility [ANOVA: F (6, 77) = 23.599, *p* < 0.01] of mice subjected to CUMS (*n* = 12, *p* < 0.01). Additionally, TrkB-shRNA infusion remarkably blocked the increasing effects of 5 mg/kg oroxylin A administration on the sucrose preference of mice subjected to CUMS [ANOVA: F (6, 77) = 18.109, *p* < 0.01] (*n* = 12, *p* < 0.01). Figure 6D illustrates the data involving CRS and TrkB-shRNA. The usage of TrkB-shRNA evidently prevented the decreasing effects of oroxylin A on the FST immobility [ANOVA: F (6, 77) = 34.226, *p* < 0.01] and TST immobility [ANOVA: F (6, 77) = 27.338, *p* < 0.01] of mice subjected to CRS (*n* = 12, *p* < 0.01). Moreover, the usage of TrkB-shRNA evidently prevented the enhancing effects of oroxylin A on the sucrose preference of mice subjected to CRS [ANOVA: F (6, 77) = 15.432, *p* < 0.01] (*n* = 12, *p* < 0.01).

In conclusion, the hippocampal BDNF-TrkB system participates in the antidepressant mechanism of oroxylin A.

## 4 Discussion

Up to date, a lot of herbal medicine has been found to produce antidepressant-like actions in rodents, such as andrographolide, tetramethylpyrazine, curcumin and ginsenoside Rg2 (Jiang et al., 2015; Seo et al., 2015; Ren et al., 2017; Zhang et al., 2019). Many researchers have an opinion that natural antidepressants are great alternatives/supplements to those monoaminergic antidepressants used in clinical practice, as natural antidepressants may have similar or even better efficacy, whereas their side effects are fewer. Oroxylin A has been demonstrated to have various pharmacological functions (Lu et al., 2016). In the present study, our findings showed that administration of oroxylin A induced notable antidepressant-like efficacy in both the CUMS and CRS models of depression. Moreover, promotion of the hippocampal BDNF signaling cascade and neurogenesis is involved in the antidepressant-like efficacy of oroxylin A. Collectively, our study indicates that oroxylin A has beneficial effects against the chronic stress-induced depressive-like symptoms, extending our knowledge of oroxylin A’s pharmacological functions and providing a novel antidepressant candidate.

The FST and TST are two tests widely adopted to screen antidepressant medications (Cryan et al., 2005; Petit-Demouliere et al., 2005). Therefore, oroxylin A was initially evaluated in the FST and TST. Our results reveal that oroxylin A notably decreased the immobility duration of mice in the two tests. It is possible that oroxylin A can promote mice locomotor activity, leading to a false-positive conclusion. To avoid this possibility, oroxylin A was then evaluated in the OFT. Our results indicate that oroxylin A produced none effects on mice locomotor activity. Thus, oroxylin A indeed has potential of being an antidepressant candidate, and moreover, its usage may avoid locomotor-related side effects which have been found for some monoaminergic antidepressants in clinical practice (fluoxetine, paroxetine, etc.). However, using only the FST and TST were not enough (Molendijk and de Kloet, 2015; de Kloet and Molendijk, 2016), and more convincing methods must be also accompanied. CUMS is a widely accepted animal model of depression which induces both behavioral and neurobiological changes in rodents, and these changes resemble clinical depression in human (Qiao et al., 2016; Antoniuk et al., 2019). CRS is similar to CUMS. The finding that repeated treatment of paroxetine notably antagonized the depressive-like behaviors induced by both CUMS and CRS indirectly support the success and effectiveness of our models. Similar to paroxetine, repeated administration of oroxylin A also produced beneficial effects against both the CUMS and CRS models, revealing that this substance may confer novel medications for the treatment of depression in the future.

Before this study, four previous reports have indicated a positive effect of oroxylin A on BDNF biosynthesis, and another one has also indicated a positive effect of oroxylin A on hippocampal neurogenesis. By using ICR mice and primary rat cortical neurons as the experimental subjects, these reports declared that treatment of oroxylin A significantly increased the levels of BDNF biosynthesis and hippocampal neurogenesis under normal condition (Lee et al., 2010; Jeon et al., 2011; Jeon et al., 2011; Kim et al., 2014). However, our western blotting and immunofluorescence data showed that although oroxylin A evidently enhanced the hippocampal BDNF expression and neurogenesis under depressive-like condition, its administration did not achieve similar efficacy under normal condition. For this discrepancy or contradiction, currently we have no reasonable explanations. It may be due to individual differences in rodents, as the experimental subjects used in our study were adult C57BL/6J mice. Moreover, it is possible that for BDNF biosynthesis and neurogenesis in C57BL/6J mice, there are some negative feedback biological mechanisms which work under normal condition but collapse under depressive-like condition, similar to biological regulation of the hypothalamic-pituitary-adrenal (HPA) axis activity. Another confusing phenomenon is that although repeated treatment of oroxylin A did not enhance the hippocampal BDNF system in C57BL/6J mice in the control group, its administration significantly decreased the immobility duration of these animals in the FST and TST. Here we speculate that under normal condition, oroxylin A administration may promote the biological activity but not expression of the hippocampal BDNF signaling cascade in C57BL/6J mice. Further in-depth molecular studies are ongoing in our groups. Anyways, by analyzing our *in vivo* and *in vitro* data together, it can be found that the greater oroxylin A’s enhancing effects on hippocampal BDNF and neurogenesis, the greater are its antidepressant-like actions. Here, we mainly studied the hippocampus region. In addition to the hippocampus, the BDNF system in other brain regions such as the prefrontal cortex (PFC) and nucleus accumbens (NAc) has also been demonstrated to correlate with depression (Reinhart et al., 2015; Koo et al., 2019). Thus, it was possible that oroxylin A produced antidepressant-like effects in mice by affecting the BDNF system in the PFC and NAc regions. To exclude this possibility, we adopted a strategy involving genetic knockdown of BDNF and TrkB in the hippocampus. As expected, the behavioral results involving BDNF-shRNA and TrkB-shRNA confirm that the hippocampal BDNF-TrkB signaling was necessary for the efficacy of oroxylin A found in this study.

As a well-known neurotrophic factor, BDNF is able to modulate many physiological processes including neuronal plasticity, neurogenesis and others. BDNF is involved in not only depression but also a lot of other neurological disorders such as Alzheimer’s disease, Parkinson’s disease and stroke (Berretta et al., 2014; Amidfar et al., 2020; Palasz et al., 2020). Thus, it will be very interesting and meaningful to study whether oroxylin A has protecting effects against these disorders in the future. Moreover, currently we could not determine that BDNF is the only antidepressant target for oroxylin A yet, since the pathophysiology of depression involves dysfunction of many other systems besides monoaminergic and neurotrophic deficiency. Oroxylin A has a lot of pharmacological functions. Previous literatures have shown that oroxylin A inhibits inflammation by influencing the activity of many molecules (NLRP3 inflammasome, ERK, PI3K/AKT, Nrf2/ARE, NF-κB, etc.) (Yao et al., 2014; Ye et al., 2014; Zhou et al., 2017; Zhang et al., 2021a). Previous literatures have also indicated that oroxylin A not only inhibits H_2_O_2_-induced oxidative stress in PC12 cells but also prevents doxorubicin-induced cardiotoxicity by activating sirt1 in mice (Han et al., 2017; Zhang et al., 2021). It should be noticed that the pathogenesis of depression is accompanied with neuroinflammation, neuronal oxidative stress and sirt1 dysfunction (Lu et al., 2018; Bhatt et al., 2020; Troubat et al., 2021). Therefore, these molecules may also contribute to the antidepressant-like effects of oroxylin A found in this study, and more antagonists/shRNAs shall be used in the next study.

In addition to pharmacological activities, toxicity is also an important criterion to evaluate a chemical with medicinal properties. Fortunately, two *in vitro* studies have demonstrated that oroxylin A has high selectivity between normal cells and cancer cells, which may be attributed to the unfolded protein response (UPR) and AKT pathways (Mu et al., 2009; Xu et al., 2012). Moreover, another *in vivo* study has reported that orally administration of 80 mg/kg (a dose far exceed 5 mg/kg) oroxylin A and 200 mg/kg imatinib notably inhibited tumor growth in mice but produced no significant changes in body weight, heart, liver, spleen and kidney (Li et al., 2015). Thus, according to current literatures (Mu et al., 2009; Xu et al., 2012; Li et al., 2015; Lu et al., 2016), oroxylin A shall possess low toxicity, but this field was limited in cancer research so far.

Overall, this is the first *in vivo* comprehensive study showing that oroxylin A has potential of being a novel antidepressant candidate, extending our knowledge of its pharmacological actions and further supporting BDNF as a useful and reliable target for antidepressants.

## Data Availability

The original contributions presented in the study are included in the article/Supplementary Material, further inquiries can be directed to the corresponding authors.

## Abbreviations

AAV, adeno-associated virus; ANOVA, one-way analysis of variance; BDNF, brain derived neurotrophic factor; CRS, chronic restraint stress; CUMS, chronic unpredictable mild stress; CREB, cAMP response element-binding protein; DCX, doublecortin; FST, forced swimming test; TrkB, tyrosine kinase B; TST, tail suspension test; OFT, open field test; SPT, sucrose preference test
