# Supplementary material for: Antidepressant-like activity of oroxylin A in mice models of depression: A behavioral and neurobiological characterization
Source: Front Pharmacol. 2022 Jul 26;13:921553. doi: 10.3389/fphar.2022.921553 (PMC9360618; doi:10.3389/fphar.2022.921553)
Supplement: Supplementary file 1 [file DataSheet1.PDF]

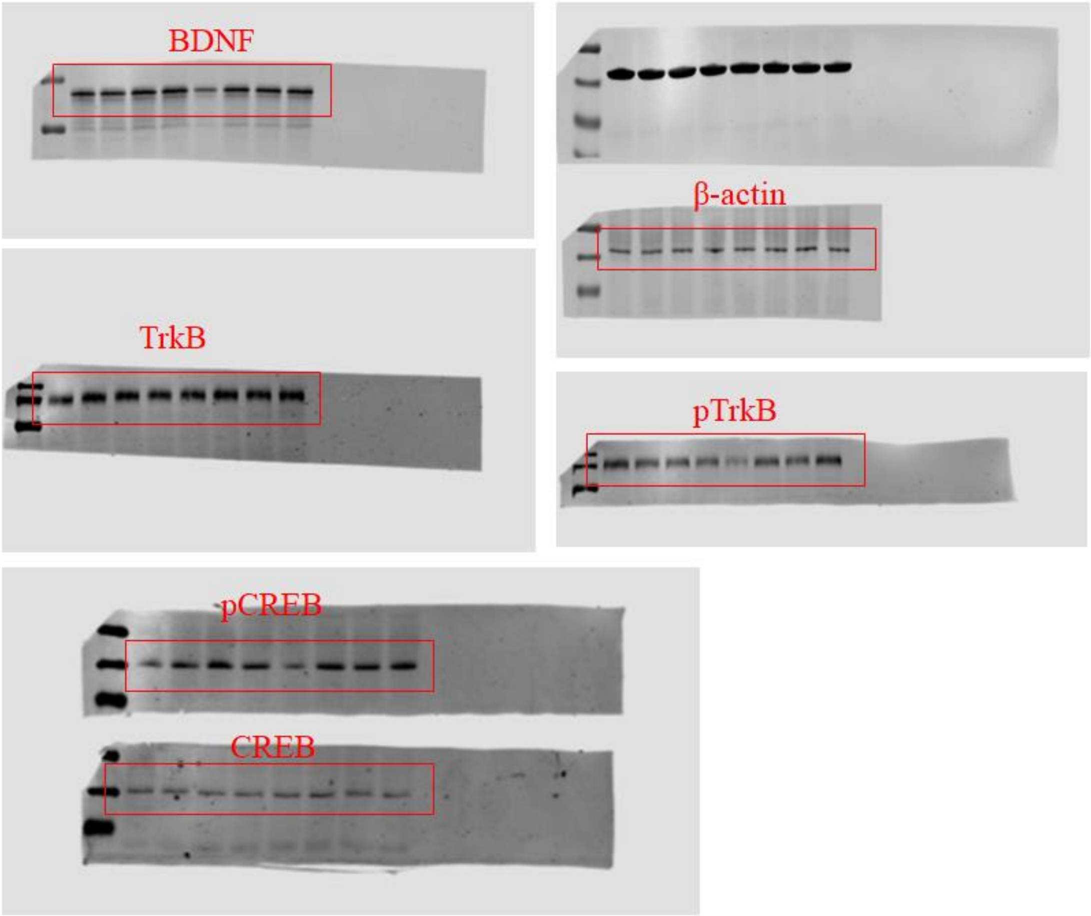

Figure S1. Original uncropped images of western blots in Figure 3A.

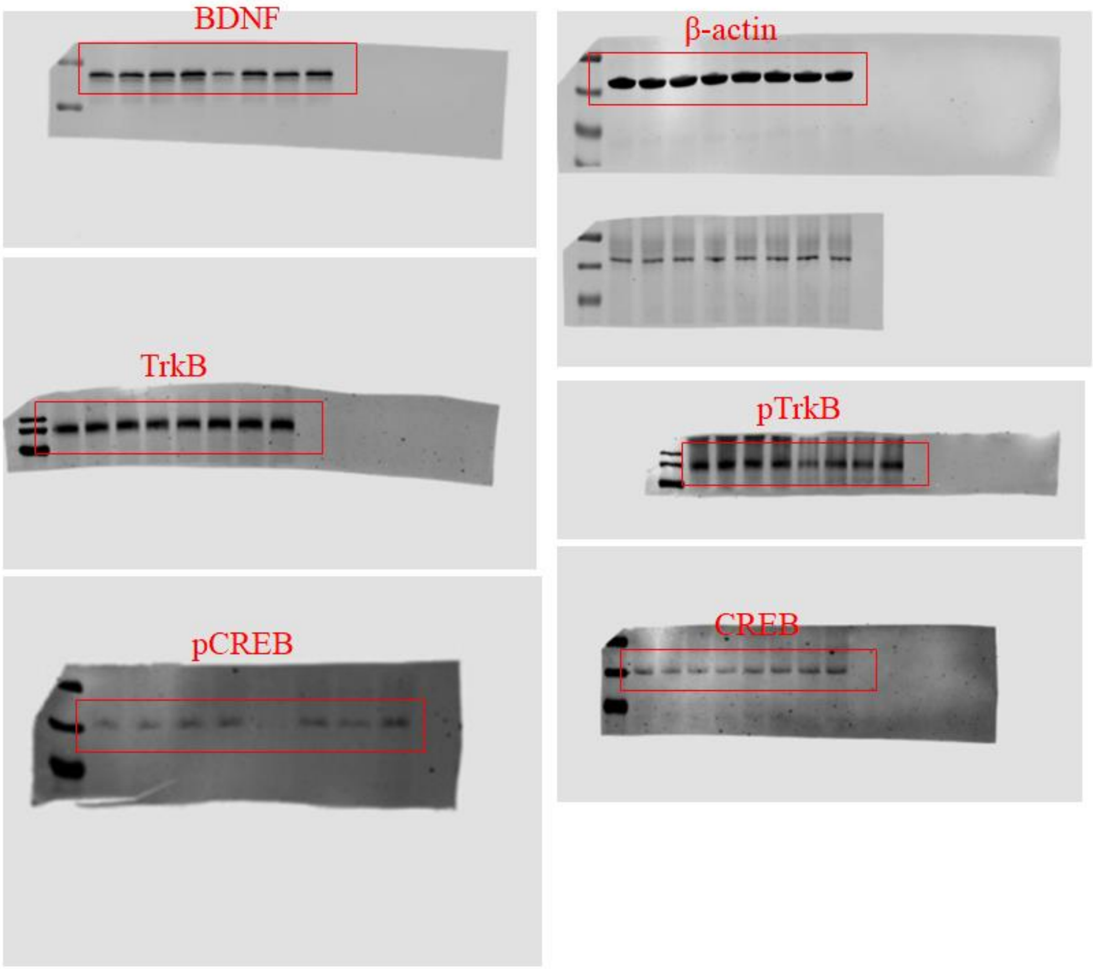

Figure S2. Original uncropped images of western blots in Figure 3B.

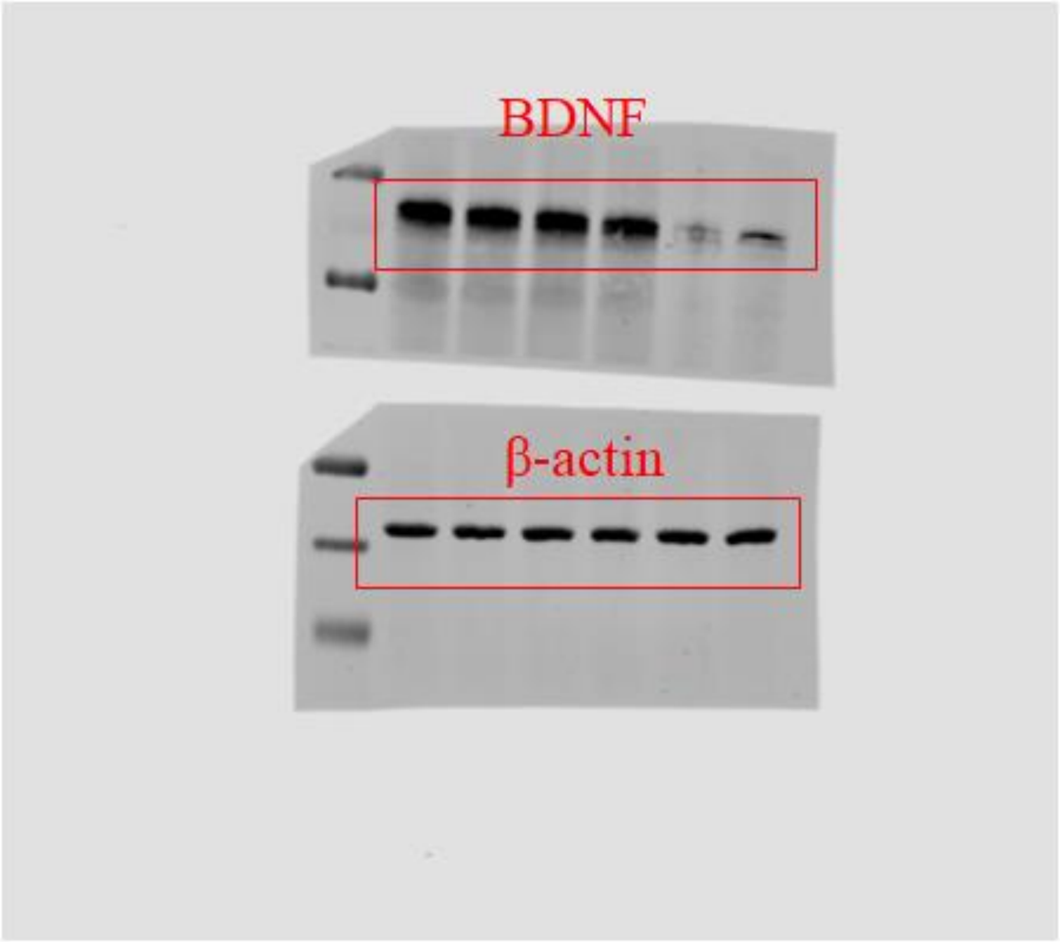

Figure S3. Original uncropped images of western blots in Figure 5A.

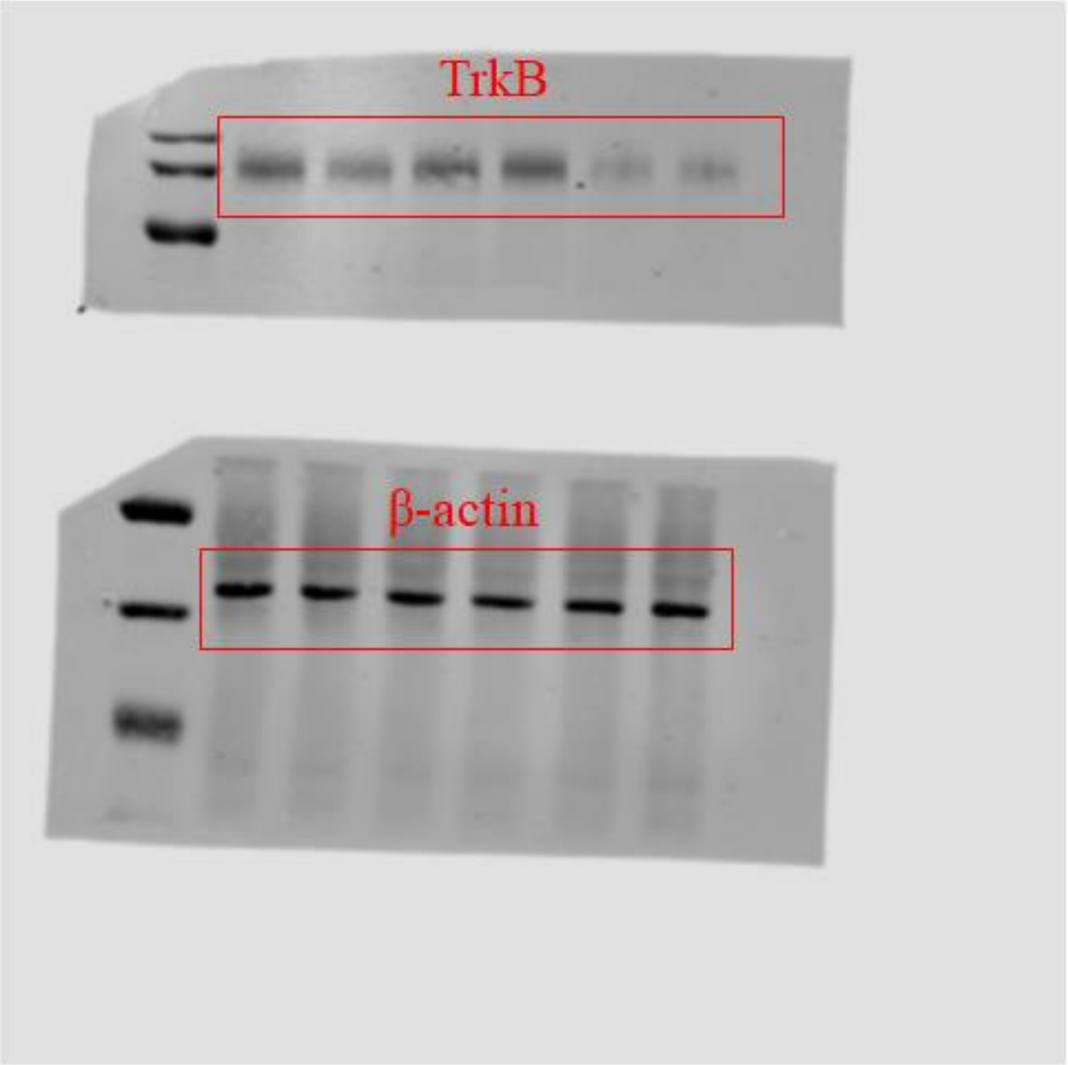

Figure S4. Original uncropped images of western blots in Figure 6A.
